# Trends change in teen pregnancy among adolescent women in Ethiopia based on Ethiopian demographic and health surveys: Multivariate decomposition analysis

**DOI:** 10.1371/journal.pone.0287460

**Published:** 2023-06-23

**Authors:** Asaye Alamneh Gebeyehu, Assefa Agegnehu Teshome, Wondwosen Teshager, Fentaw Teshome, Mulu Tiruneh, Anteneh Mengist Dessie, Denekew Tenaw Anely, Aragaw Tesfaw, Dejen Gedamu Damtie, Chalachew Yenew

**Affiliations:** 1 Department of Public Health, College of Health Sciences, Debre Tabor University, Debre Tabor, Ethiopia; 2 Department of Biomedical Science, College of health sciences, Debre Tabor University, Debre Tabor, Ethiopia; Maroof International Hospital, PAKISTAN

## Abstract

**Background:**

Teenage pregnancy may adversely affect their health, economic, and social life. Evidence shows that no studies in Ethiopia used decomposition analysis to identify factors for the trend change in teen pregnancy. Therefore, this study aimed to examine the trends and identify contributing factors to teen pregnancy in Ethiopia using multivariate decomposition analysis.

**Method:**

We obtained the data on adolescent women from three Ethiopian Demographic and Health Surveys. A weighted sample of 3266 in 2005, 4009 in 2011, and 3381 teenagers in 2016 were involved in this study. Statistical analysis was performed using STATA 14. Multivariate decomposition analysis was used to identify contributing factors to the change in teen pregnancy. The coefficient value with a 95% confidence interval was used to identify significant factors associated with teen pregnancy.

**Results:**

The prevalence of teen pregnancy in Ethiopia decreased significantly from 15.9% [95% CI: 14.3, 17.7] in 2005 to 12.5% [95% CI: 10.8, 14.3] in 2016. Multivariate decomposition analysis showed that approximately 83% of the overall change in teen pregnancy over time was due to differences in women’s composition. Age, marital status, education status, working status, contraceptive use, and sexual status before age 18 were statistically significant contributing factors to the decline in teen pregnancy over time.

**Conclusion:**

The prevalence of teen pregnancy in Ethiopia decreased significantly over time. The overall decline in teenage pregnancy is due to differences in population composition. Public health interventions should focus on changing cultural norms or attitudes regarding early marriage and pregnancy within religious leaders and uneducated communities.

## Background

The World Health Organization (WHO) defines adolescence as a transitional period from childhood to adulthood, usually between 10–19 years of age [1–3]. Globally, more than half (50%) of the population are young people aged under 25 years, and 85% of the world’s adolescent population lives in developing countries [4]. An estimated 21 million girls aged 15–19 years become pregnant every year, and 16 million of them give birth [5]; 95% of all babies born to adolescent mothers occur in developing countries, particularly Sub-Saharan Africa [6]. Each year, more than 3 million adolescents undergo unsafe abortions in the world, and girls between 15–19 years of age are more likely to die during pregnancy and childbirth than women over 20 [7].

Teen pregnancy has several adverse effects on their health, economy, and social life [8]. It is a leading cause of morbidity and mortality [9, 10]. Teenagers also suffer from serious health problems of fistula, unsafe abortion, eclampsia, induced hypertension, puerperal endometritis, anemia, and postpartum hemorrhage [11, 12]. Annually, an estimated 68,000 deaths from adolescent mothers have experienced unsafe abortions [10]. Babies born to teenage mothers are 60% more likely to die before the first birthday than those to older mothers, and stillbirths and newborn deaths are 50% higher among babies born to teen mothers in the first week of birth [10]. Babies born to teens mothers are highly at risk of low birth weight, preterm birth, and neonatal mortality [13].

Ethiopia has the highest teenage pregnancy rate among sub-Saharan African countries. According to the 2016 EDHS report, 17.4% of mothers die from pregnancy-related complications [14]. Teenage mothers and their children are deprived of essential healthcare services due to a lack of access to healthcare, skilled delivery services, or delays in access to antenatal care (ANC) [15]. The Ethiopian government has formulated laws and regulations to mitigate teen pregnancy by prohibiting the law of illegal marriage before 18 years of age [8, 16]. Hence, early marriage and pregnancy make it difficult to reach the Sustainable Development Goals (SDGs) related to health, economy, and social life [17].

Previous studies across the countries have identified several contributing factors to teenage pregnancy. These studies include age [18–20], marital status [19, 21], educational status [21–23], contraceptive use, early sexual intercourse [21, 24–26], respondent’s working status [20, 21], and wealth index [27, 28] were associated with teen pregnancy.

Despite increased efforts in health care services, teen pregnancy remains high in Ethiopia [14, 29]. Previous studies were done in some local areas and used only specific datasets [18, 19, 22, 30]; it was difficult to determine the trend and identify contributing factors to the change in teen pregnancy over time in Ethiopia. Evidence shows that no studies in Ethiopia used decomposition analysis to analyze trend changes in pregnancy among teenagers based on the national dataset. Therefore, the objective of this study is to examine the trend and identify contributing factors to the change in teen pregnancy over time using multivariate decomposition analysis. These findings will help policymakers and designers to focus on improving girls’ health education and changing their cultural attitudes towards early marriage and teen pregnancy.

## Methods

### Study areas and setting

This study was conducted in Ethiopia. Ethiopia is one of the East African countries, with an estimated population of more than 112 million [31]. Nationally, nearly half (47%) of females belong to 15–19 years of age [32].

Ethiopia is an African country with an agriculture-dependent economy, and 79% of the population lives in rural areas [33]. The country has nine geographical regions; Tigray, Afar, Amhara, Oromiya, Somali, Benshangul-Gumuz, Southern Nations Nationalities and People (SNNP), Gambela, and Harari. The two administrative cities are Addis Ababa and Dire Dawa. According to the 2007 population census, each smaller unit (Kebele) was subdivided into enumeration areas or clusters, easy-to-perform census studies [32].

### Data source, study design, and study population

We utilized data on women from 2005, 2011, and 2016 Ethiopia Demographic and Health Surveys. This study was a cross-sectional data analysis of the Ethiopian Demographic and Health Survey (EHDS). The EDHS uses a two-stage stratified sampling technique to select the sample size. According to the 2007 PHC, the sample was selected using the two stages. In the first stage, a total of 540 clusters (395 in rural areas and 145 in urban areas) for EDHS 2005, 624 clusters (437 in rural areas and 187 in urban areas) for EDHS 2011, and 645 clusters (443 in rural areas and 220 in urban areas) for EDHS 2016 were selected with proportional allocation to cluster size.

In the second stage, household listing operations were completed in each selected cluster; and the list of households served as a sampling frame for selecting household sizes in the second stage. On average, 27 to 32 households per cluster were randomly selected proportional to the cluster size. From all eligible households, 14070 reproductive-aged with a 96% response rate in 2005, 16515 women with a 95% response rate in 2011, and 15683 women with a 95% response rate in 2016 completed interviews. The study used data from women’s questionnaires, particularly among women aged between 15 and 19 from the survey. Moreover, detailed information about sampling methods, data validation, and quality assurance was available in DHS measures [2, 34, 35].

### Study variables

#### Dependent variable

The outcome variable was teenage pregnancy, which consists of currently pregnant at data collection time, having a child, and terminated pregnancy included in this study. It is referring the percentage of teenagers who are mothers, pregnant with their first child, and have begun childbearing. The response variable is a binary outcome variable present by a random variable Y_i_ for the ith teenager, which has two possible values coded as "1" if the teenager experienced the pregnancy before age 20 and "0" if not experienced the pregnancy before 20 years.

#### Independent variables

The independent variables included in this study were age, place of residence, marital status, educational status, respondents’ working status, sex of the household head, age of the household head, early sexual intercourse, mass media exposure, wealth index, and contraceptive use. Relevant independent variables were obtained from the EDHS datasets.

#### Data management and analysis

Data cleaning, coding, and analysis were performed using Stata 14 software. Sampling probability is vital to handle sampling bias and restore the representative sample. We carried out cross-tabulation and summary statistics to describe the study population. Multicollinearity between two variables can be checked using the variance inflation factor (VIF). If the VIF value is usually less than 10%, there is no multicollinearity assumption between independent variables. Data on adolescent women for this study were obtained from the Women’s Recode (IR file) datasets, with a weighted sample size of 3266, 4009, and 3881 teenagers from 2005, 2011, and 2016 EDHS included in it. We appended datasets together to perform trend and decomposition analysis after extracting relevant variables from EDHS 2005, 2011, and 2016. The coefficient value with a 95% confidence interval was used to identify significant independent variables associated with teenage pregnancy.

### Trend and decomposition analysis

The trend period was divided into three phases such as; the first phase (2005–2011), the second phase (2011–2016), and the third phase (2005–2016) to see the differences in the prevalence of teen pregnancy over time-based on different selected characteristics of women. The trend was assessed using descriptive analysis stratified by various selected explanatory variables and examined separately for each phase.

Multivariate decomposition analysis of the change in teen pregnancy was used to identify the major contributing factors to the change in the percentage of teen pregnancy over the study period. The decomposition analysis targeted how the change in teen pregnancy responds to the differences in respondent or women’s characteristics and how these variables shape the changes across the survey conducted at different times. It is a regression analysis of the differences in the percentage of teen pregnancy between EDHS 2005 and EDHS 2016. The purpose of using the multivariate decomposition analysis is to identify the potential source of the differences in the percentage of teen pregnancy in the last decades of ten years. The multivariate decomposition analysis for the non-linear response model uses the output of logistic regression analysis because it is a binary outcome to divide the observed difference in the percentage of teen pregnancy between the surveys into the components. The differences in the composition of the population (Endowment) and the difference in the effect of the characteristics (Coefficient) are essential to identify the factors contributing to the change in teen pregnancy prevalence over time. The change in teen pregnancy prevalence is additively decomposed into women’s composition change between the surveys (Endowment) and the difference in the effect of selected independent variables (Coefficient).

The recent EDHS 2016 and baseline EDHS 2005 surveys were denoted by A and B, respectively.

For logistic regression, the log-odds or logit of teen pregnancy can be decomposed as:

$$\begin{array}{l} logit(A)-logit(B)=F(X_{A}\beta_{A})-F(X_{B}\beta_{B})\\ \;=\underset{E}{\underset{︸}{\left[F(X_{A}\beta_{A})-F(X_{B}\beta_{A})\right]}}+\underset{C}{\underset{︸}{\left[F(X_{B}\beta_{A})-F(X_{B}\beta_{B})\right]}} \end{array}$$

Where: E represents endowments explained by characteristics, and C represents coefficients not explained.

We can rewrite the above equation as follow:

$$logit(A)-logit(B)=[\beta_{0A}-\beta_{0B})+\sum X_{ijB}*[\beta_{ijA}-\beta_{ijB}]+\sum \beta_{ijB}*[X_{ijA}-X_{ijB}]$$

Where; *β*_0*B*_ is the intercept in the regression equation for EDHS 2005,

*β*_0*A*_ is the intercept in the regression equation for EDHS 2016,

*β*_*ijB*_ is the coefficient of the *j*^*th*^ category of the *i*^*th*^ determinant in EDHS 2005,

*β*_*ijA*_ is the coefficient of the *j*^*th*^ category of the *i*^*th*^ determinant in EDHS 2016,

*X*_*ijB*_ is the proportion of the *j*^*th*^ category of the *i*^*th*^ determinant in EDHS 2005, and

*X*_*ijA*_ is the proportion of the *j*^*th*^ category of the *i*^*th*^ determinant in EDHS 2016

Currently developed multivariate logistic decomposition analysis for the non-linear response model used for the decomposition analysis of teen pregnancy using **mvdcmp** STATA package [36].

### Ethical consideration

An authorization letter was obtained from the online publicly available DHS measure program to access and download the datasets by describing the purpose of the study and used only for this study. This study used the secondary data of publicly available survey data from the Measure DHS program since we did not need ethical approval. We downloaded the datasets for this current study from the website http://www.dhsprogram.com. Datasets obtained from the Measure DHS program are kept confidential.

## Results

### Background characteristics of the study population

A total of 32666, 4009, and 3881 adolescent women were included in the study. More than a fifth-sixth (84.1%) of the teenagers have not experienced pregnancy, and 12.9% in 2016 were pregnant. Based on the age of teenagers, more than sixty percent (60%) of all study participants were in the age group 15 to 17 years (Table 1). According to the sex of the household head, more than three-quarters (75%) of the households were led by males. Rural and unmarried teenagers comprised more than 73% in all three consecutive surveys.

**Table 1:** Background characteristics of the study population in 2005, 2011, and 2016 EDHS.

Variables	Categories	EDHS2005	EDHS2011	EDHS2016
		N = 3266 (%)	N = 4009 (%)	N = 3881 (%)
Teen pregnancy	No	2744 (84.1)	3521 (87.9)	2960 (87.5)
	Yes	522 (15.9)	488 (12.1)	421 (12.5)
Age of teenagers	15–17	1953 (59.8)	2454 (62.2)	2050 (60.6)
	18–19	1313 (40.2)	1555 (38.8)	1331 (39.4)
Sex of household head	Male	2500 (76.5)	2922 (78.9)	2499 (73.9)
	Female	766 (23.5)	1089 (21.1)	882 (26.1)
Place of residence	Urban	704 (21.5)	1042 (26.0)	805 (23.8)
	Rural	2562 (78.5)	2967 (74.0)	2576 (76.2)
Marital status	Single	2394 (73.3)	3087 (77.0)	2642 (78.1)
	Married/living together	711 (21.8)	765 (19.1)	888 (17.4)
	Widowed/divorced/separated	160 (4.9)	157 (2.9)	151 (4.5)
Religion	Orthodox	1703 (52.2)	2022 (50.4)	1446 (42.2)
	Protestant	606 (18.5)	833 (20.8)	847 (25.0)
	Muslim	859 (26.3)	1075 (26.8)	1064 (31.5)
	Other	98 (3.0)	78 (2.0)	44 (1.3)
Educational status	No education	1308 (40.0)	695 (17.3)	469 (13.9)
	Primary	1423 (43.6)	2813 (70.2)	2148 (63.5)
	Secondary & above	535 (16.4)	501 (12.5)	765 (22.6)
Wealth index	Poor	1013 (31.0)	1382 (34.5)	1036 (30.6)
	Middle	627 (19.2)	687 (17.1)	668 (18.9)
	Rich	1625 (49.8)	1940 (48.4)	1708 (50.5)
Respondent’s working status	Yes	908 (27.8)	2061 (51.4)	1253 (37.1)
	No	2358 (72.2)	1948 (48.6)	2128 (62.9)
Early sexual intercourse before 18	Yes	805 (24.6)	815 (20.3)	741 (21.9)
	No	2461 (75.4)	3194 (79.7)	2640 (78.1)
Contraceptive use	Yes	84 (2.5)	214 (5.3)	254 (7.5)
	No	3182 (97.5)	3795 (94.7)	3127 (92.5)
Media exposure	Yes	1767 (54.1)	2931 (73.1)	1616 (47.8)
	No	1499 (45.9)	1078 (26.9)	1765 (52.2)
Age of household head	<30	637 (19.6)	766 (19.1)	551 (16.3)
	30–44	728 (22.3)	1029 (25.7)	796 (23.5)
	45–54	852 (26.1)	966 (24.1)	845 (25.0)
	55–64	701 (21.5)	880 (22.0)	838 (24.8)
	65+	345 (10.6)	368 (9.2)	351 (10.4)

Regarding maternal educational level, the proportion of teenagers with no formal education revealed a high decline from 40% in 2005 to 13.9% in 2016. However, the proportion of adolescent women with primary education increased from 43.6% in 2005 to 63.5% in 2016. Based on the place of residence, more than 74% of the teenagers were rural residents. The proportion of Orthodox Christians decreased from 52.2% in 2005 to 42.2% in 2016. But Protestants and Muslims increased from 18.5% and 26.3% to 25% and 31.5%, respectively.

Across the three surveys, the proportion of women who experienced early sexual intercourse (before 18 years old) decreased from 24.6% in 2005 to 21.9% in 2016. In the three consecutive surveys, 92% of teenagers did not use contraception. Approximately thirty-one (31%) of adolescent women from all surveys were poor (Table 1).

### Prevalence of teenage pregnancy in Ethiopia

**Fig 1:**
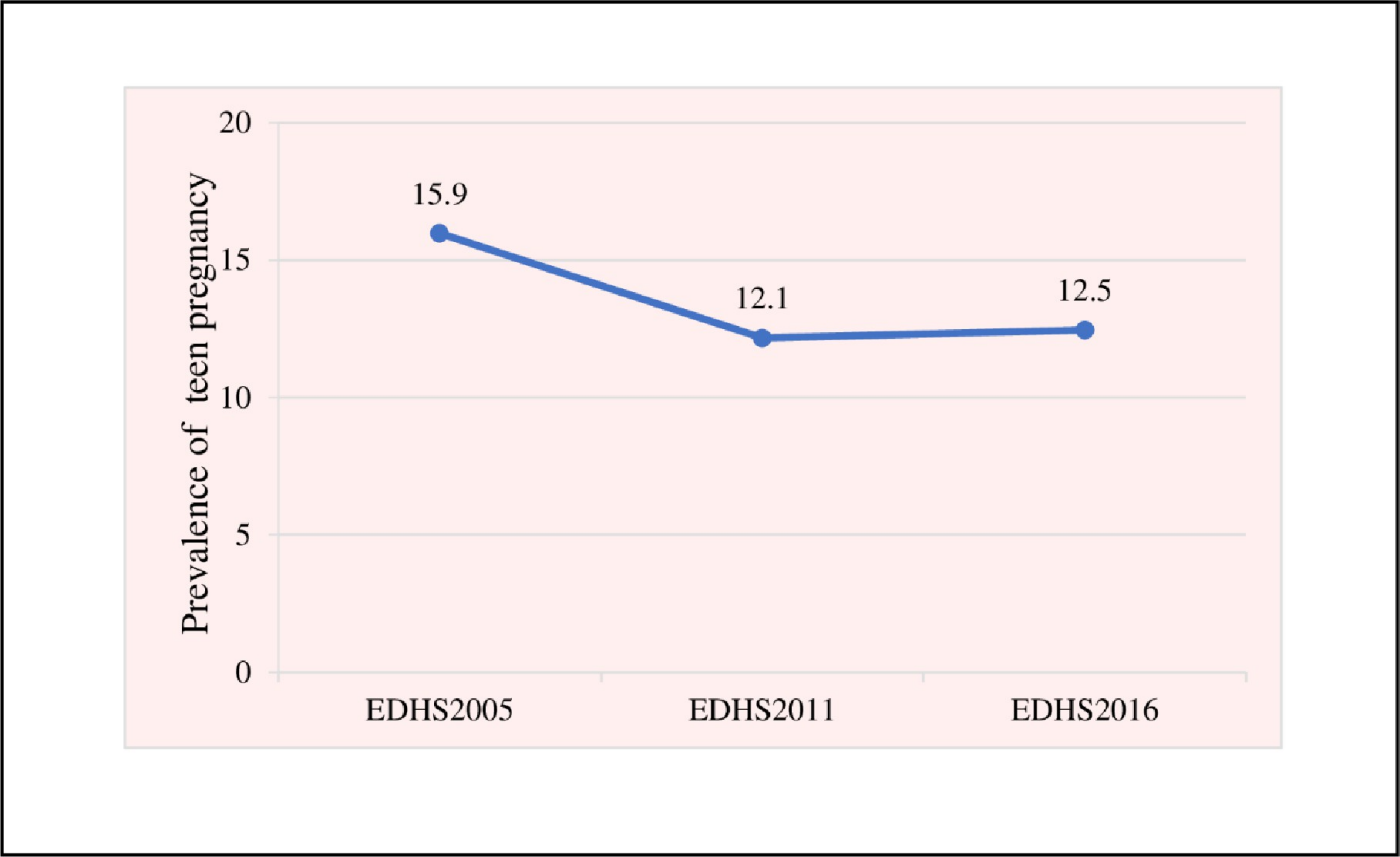
The trends of teen pregnancy in Ethiopia from 2005 to 2016.

The prevalence of pregnancy among teenagers in Ethiopia decreased from 15.9% in 2005 to 12.5% in 2016 in the last decades. The trends of teen pregnancy over the study periods (2005–2016) were divided into three phases; 2005–2011, 2011–2016, and 2005–2016 to see the differences in teen pregnancy over the study periods and the sources for the change in teen pregnancy. The first phase (2005–2011) showed a 3.8% point decrease in the level of teenage pregnancy, while the second phase indicated a 0.5% point increase in teen pregnancy between 2011 and 2016. The overall third phase between 2005 and 2016 displayed a 3.4% point change decrease in teen pregnancy. The prevalence of teen pregnancy declined from 15.9% [95% CI: 14.3, 17.7] in 2005 to 12.5% [95% CI: 10.8, 14.3] in 2016 (p-value <0.001), with a 3.4% overall point change (Fig 1).

### Trends in the prevalence of teen pregnancy by selected characteristics

Trends of pregnancy among teenagers revealed the variation based on different characteristics. The percentage point change in teen pregnancy decreased in many categories of the variables in each phase (Table 2).

**Table 2:** Trends in teen pregnancy prevalence among women by selected characteristics 2005, 2011, and 2016 Ethiopia Demographic and Health Surveys.

Characteristics	EDHS2005	EDHS2011	EDHS2016	Percentage point difference in teen pregnancy prevalence		
	Percentage point (%)			Phase1	Phase2	Phase3
				2011–2005	2016–2011	2016–2005
Teen pregnancy	15.9	12.1	12.5	-3.8	0.5	-3.4
Age of teenagers						
15–17	7.5	4.1	6.3	-3.4	2.2	-1.2
18–19	28.6	24.8	22.0	-3.8	-2.8	-6.6
Sex of household head						
Male	18.4	13.5	13.7	-4.9	.2	-4.7
Female	8.1	8.7	9.1	.6	.4	1.0
Place of residence						
Urban	6.4	4.3	5.5	-2.1	1.2	-.9
Rural	18.6	14.9	14.7	-3.7	-.2	-3.9
Marital status						
Single	0.5	0.3	0.6	-.2	.3	.1
Married/living together	66.0	57.3	60.3	-8.7	3.0	-5.7
Widowed/divorced/separated	25.7	25.9	33.7	.2	7.8	8.0
Religious						
Orthodox	15.1	11.2	7.9	-3.9	-3.3	-7.2
Protestant	11.0	11.9	10.2	.9	-1.7	-.8
Muslim	22.0	14.1	20.3	-7.9	6.2	-1.7
Other	9.3	15.0	15.5	5.7	.5	6.2
Educational status						
No education	27.8	32.4	28.0	4.6	-4.4	.2
Primary	10.1	8.6	12.0	-1.5	3.4	1.9
Secondary & above	2.8	4.3	4.2	1.5	-.1	1.4
Wealth index						
Poor	21.1	16.7	20.2	-4.4	3.5	-.9
Middle	18.3	16.2	15.0	-1.9	-1.2	-3.3
Rich	11.9	7.5	6.8	-4.4	-.7	-5.1
Respondent’s working status						
Yes	11.4	10.8	12.4	-.6	1.6	1.0
No	17.7	13.4	12.5	-4.3	-.9	-5.2
Early sexual intercourse						
Yes	59.4	53.7	54.4	-5.7	.7	-5.0
No	1.8	1.6	0.7	-.2	-.9	-1.1
Contraceptive use						
Yes	49.5	37.8	35.7	-11.7	-2.1	-13.8
No	15.1	10.7	10.5	-4.4	-.2	-4.6
Media exposure						
Yes	13.3	10.3	9.1	-3.0	-1.2	-4.2
No	20.3	17.3	15.5	-3.0	-1.8	-4.8
Region						
Large central	16.6	12.4	12.8	-4.2	0.4	-3.8
Small peripheral	19.3	19.0	17.9	-.3	-1.1	-1.4
Metropolitan	6.0	4.0	5.0	-2.0	1.0	-1.0
Age of household head						
<30	52.7	39.0	41.5	-13.7	2.5	-11.2
34–44	11.6	7.4	7.8	-4.2	.4	-3.8
45–54	3.8	2.4	6.7	-1.4	4.3	2.9
54–64	5.8	7.0	5.1	1.2	-1.9	-.7
65+	8.3	7.8	8.9	-.5	1.1	.6

The prevalence of teen pregnancy declined by a 3.9% point of change among rural teenagers during the third phase (2005–2016). Based on geographical regions, a decrement in teen pregnancy prevalence showed in the last period (2005–2016) (Table 2). The highest percentage point difference in teen pregnancy prevalence was 3.8 in the large central region (Tigray, Amhara, Oromia, and SNNPs); however, the least point change in the percentage of teen pregnancy was 1% in the metropolitan geographical regions (Harari, Addis Ababa, and Dire Dawa) (Table 2).

According to educational status, there was an increase in the percentage of pregnancy among teenagers with primary and secondary or above education in the last phase (2005–2016) by 1.9% and 1.4%, respectively. Teenagers who were married/or living together with their partners and Orthodox religious followers revealed a decrement in the prevalence of teen pregnancy with 5.7% and 7.2% point change, respectively. Additionally, women who had used contraceptive methods showed a decrement in teen pregnancy prevalence with a 13.8% point change (Table 2).

## Decomposition analysis

### Decomposition analysis of teen pregnancy in Ethiopia, 2005–2016

A general trend decomposition analysis showed a significant decrease in the prevalence of teen pregnancy in Ethiopia. The decomposition results revealed that the decline in teen pregnancy prevalence over the study periods was explained only by the difference in the selected women’s characteristics between the two survey points. However, the decline in teen pregnancy prevalence due to the differences in the effect of the selected independent variables was not statistically significant (Table 3).

**Table 3:** Overall decomposition of the change in teen pregnancy among teenagers in Ethiopia 2005–2016.

Teenage pregnancy	Coef.	[95% conf. interval]	Pct
Characteristics (E)	-.047891	-.066444 -.029337	82.95
Coefficient (C)	-.02259	-.087969 .042789	17.05
Residual	-.070481	-.13194 -.0090207	

### Differences due to characteristics (Endowment)

Multivariate decomposition analysis results revealed that the overall decline in teen pregnancy was due to the difference in characteristics (the differences in the composition of women’s selected variables) between the two survey points (Table 3). Age, marital status, employment status, education level, contraceptive use, and having sex before 18 years of age contributed to the change in the prevalence of teen pregnancy (Table 4).

**Table 4:** Detailed decomposition analysis of change in teenage pregnancy in Ethiopia 2005–2016.

Teen pregnancy Variables	Difference due to Characteristics (E) Coef Pct	Difference due to Coefficient (C) Coef Pct
Age of teenagers		
15–17^®^		
18–19	-.00084972* (-.0014561 -.00024334) 1.21	-.011049 (-.030451 .0083522) 15.67
Sex of household head		
Male^®^		
Female	.0014713 (-.00091599 .0038585) -2.09	.0044977 (-.0058855 .014881) -6.38
Place of residence		
Urban^®^		
Rural	-.00054377 (-.0031155 .002028) 0 .77	.0069536 (-.030967 .044875) -9.86
Marital status		
Single^®^		
Married/living together	-.014013* (-.019058 -.0089678) 19.88	.0023226 (-.010712 .015358) -3.29
Widowed/divorced/separated	-.00058586* (-.0011468 -.000024935) .83	.0018718 (-.0021875 .0059311) -2.65
Educational status		
No education	-.025518 (-.055406 .0043699) 36.21	-.00029428 (-.023754 .023165) .42
Primary education	.016266* (.0043708 .036903) -23.1	-.010686 (-.039389 .018016) 15.2
Secondary & above ^®^		
Wealth index		
Poor^®^		
Middle	-.000079961 (-.00039821 .00023828) .11	.0027491 (-.0050253 .010524) -3.9
Rich	.00016268 (-.00044486 .00077021) -.23	-.0031369 (-.021862 .015588) 4.45
Working status		
Yes^®^		
No	-.0093764* (-.017614 -.0011385) 13.3	.004034 (-.0061784 .014247) -5.72
Contraceptive use		
Yes^®^		
No	-.007223* (-.011196 -.0032496) 10.25	.015051 (-.020255 .050357) -21.35
Early sex intercourse		
Yes	-.0073953* (-.009919 -.0048716) 10.49	.0085279 (-.0071868 .024243) -12.1
No^®^		
Media exposure		
Yes^®^		
No	-.00020598 (-.0048354 .0044234) .29	-.00068561 (-.015039 .013668) .97

®: Reference category; *: Significant; Pct: Percentage; Coef: Coefficient

Multivariate decomposition analysis showed that the age, marital status, respondents’ working status, educational status, contraceptive use, and early sexual intercourse were statistically significant variables for the change in teen pregnancy prevalence. The decrement in the proportion of women aged between 18 and 19 in the sampled population revealed a significant positive 1.21% contribution to the decline in the prevalence of teen pregnancy. A decrease in the composition of women who were married/or living together with partners in the sampled population showed a significant inverse effect on the prevalence change in teen pregnancy by 19.88%. The decline in widowed/divorced/separated population composition contributed a positive 0.83% to the decline in teen pregnancy prevalence (Table 4). An increase in the proportion of teenagers with primary education had a statistically significant effect on the change in teenage pregnancy. The compositional changes of women with primary education over the study periods (from 2005 to 2016) showed a significant 23.1% negative contribution to the change in teen pregnancy. A decrease in the proportion of respondents who had no job or were not working over the study periods (from 2005 to 2016) showed a significant 13.3% positive contribution to the decline in teen pregnancy (Table 4). The decrease in the proportion of women who did not use contraceptive methods contributed to a significant decline in the prevalence of teenage pregnancy, contributing to a 10.25% point change. Moreover, teenagers who had not experienced early sexual intercourse before age 18 in the sample showed a significant positive 10.49% contribution to the decrease in the prevalence of teen pregnancy (Table 4).

Notice that the negative signs on the percentage changes in teen pregnancy show that compositional changes in characteristics have a negative or reversal effect on teen pregnancy.

## Discussion

Teenage pregnancy is a predominant public health challenge in the well-being of one’s health and childbirth outcomes. This study assessed the trends and the contributing factors for the change in teen pregnancy in Ethiopia using the EDHS 2005–2016. The trend changes in teen pregnancy prevalence declined significantly from 15.9% in 2000 to 12.5% in 2016. This finding is consistent with studies conducted in sub-Saharan Africa and Ethiopia, which have shown a declining trend in teenage pregnancy [37, 38]. The possible reason could be the government and non-government organizations have taken various measures or interventions to create a favorable environment for teenagers to access reproductive health care services and to change cultural attitudes within the communities towards early marriage. Healthcare providers offer health education in the communities to change their perception of early marriage and pregnancy practices.

In the decomposition analysis, the prevalence of teen pregnancy declined significantly over the study periods. Therefore, understanding the potential sources of variation in teenage pregnancy has public health implications for identifying factors to change in teen pregnancy prevalence and evaluating and designing strategies for reproductive health care services. Approximately 83% of the overall decline in teen pregnancy over the last ten years was due to the differences in women’s composition over the study periods; the difference due to the differences in the effect of explanatory variables (behavioral changes) was not significant.

A decrement in the proportion of adolescent women aged between 18 and 19 from 20005 to 2016 showed a significant 1.2% negative effect on the decline in teen pregnancy. Besides, as the age of women has increased, there is an increased likelihood of experiencing pregnancy before age 18. This finding is compatible with the studies conducted in Ethiopia and Nigeria [19, 20, 22]. The reason could be their reproductive life increases with age since they are more exposed to biological and social factors, specifically marriage [19]. The possible explanation might be most teens mothers were more active in sexual intercourse and marriage due to their family’s history. It implies they are tending a greater risk of pregnancy and childbirth-related complications.

A decrement in the composition of women who were not working jobs revealed a significant positive effect on the change in the prevalence of teen pregnancy, contributing to 13.3% of the changes. This finding is consistent with a previous study in Ethiopia, Nigeria, and East Africa, in which women who had no job were more likely to experience teen pregnancy [21, 24, 37]. The possible reason might be women who had no work or were unemployed in work activities are to be economically dependent, at high risk of school dropout rate, and are prone to harmful practices in early marriage and pregnancy. This result reinforces the contribution of working jobs to achieve positive health outcomes.

A decrement in the proportion of women who did not use contraceptive methods showed a significant 10.25 positive contribution to the decline in the prevalence of teen pregnancy. This finding is consistent with prior studies conducted in Ethiopia and Baltimore [22, 39]. The reason might be Teenagers may be unable to argue the use of condoms or partners’ fidelity, leaving them at higher risk of STDs and unintended pregnancy [40, 41]. They are embarrassed to find information about where to find contraceptive methods and how to use these methods during sexual intercourse since they lack access to basic health care and reproductive health care services [40]. Teenagers who become pregnant before reaching 18 years have a higher risk of mother and infant mortality due to pregnancy-related complications. Additionally, unprotected sex increases the distribution of sexually transmitted infectious diseases like HIV and other health-related problems.

The composition changes of teenagers who had experienced early sexual intercourse before age 18 showed positive contributions to the decline in teen pregnancy prevalence. A decrement in the composition of women who experienced early sexual intercourse revealed a significant 10.49% positive contribution to a decrease in teen pregnancy prevalence. This finding is consistent with prior studies in Ethiopia, Baltimore, and Johannesburg [38, 39], in which early sexual initiation is more associated with early pregnancy due to a lack of adequate information about reproductive health care services and unmet need for contraception [42]. Besides, forced sexual intercourse is another challenge that predisposes teenagers to premarital sex harassment causing early pregnancy [30]. This result indicated that experiencing early sexual intercourse is a major leading cause of early pregnancy, fistula, unsafe abortion, and death.

An increment in the proportion of women with primary education showed a statistically significant effect on the change in teen pregnancy. This study is compatible with other studies done in Ethiopia [38, 43], Nigeria [20], South Asia [44], and Philippes [26], in which girls in school are less likely to engage in sexual activity and become pregnant at an early age. The explanation is that women with formal education may have better knowledge to refuse early marriage and pregnancy at a young age and may understand the lifelong negative consequences on their health and children. In addition, educated women could know the benefit of accessing sexual health care services to delay pregnancy, control unsafe abortion, and avoid unintended pregnancy. Staying longer in school may delay the onset of getting marriage and childbirth.

A decrease in the composition of women who were married/or living together with partners over time showed a significant effect on the change in teen pregnancy. This finding is consistent with other studies conducted in Ethiopia and Japan [21, 23]. The reason might be women or teenagers could have low involvement in decision-making to utilize reproductive health care services, particularly any contraceptive method to prevent early pregnancy. The Ethiopian government has formulated rules and regulations to protect against the harmful practice of early marriage and control sexual and reproductive health problems.

## Strengths and limitations of the study

The strength of this study was the use of large-scale data sets for obtaining appropriate weighted sample size. We also used decomposition analysis to analyze the contributing factors for the change in teen pregnancy. In addition, consider sampling methods to adjust the non-response rate and restore the nationally representative sample.

As a limitation of this study, this secondary data did not measure relevant variables during data collection time; religious beliefs towards marriage, family structure, perception of family planning, decision-making involvement, psychological, social support, and cultural norms. Furthermore, our model is limited to EDHS datasets to explain the difference in the response variable. We recommend future researchers use another alternative method to the decomposition analysis.

## Conclusion

Teen pregnancy prevalence decreased significantly in the last decades. However, the pregnancy rate among adolescent women is still high in Ethiopia. Complications during pregnancy and childbirth are leading to the death of mothers and infants. Approximately five-sixth of the overall decline in teen pregnancy prevalence was only due to the difference in the composition of women’s characteristics. The significant differences due to the compositional changes in women’s selected factors were age, educational level, respondents’ working status, contraceptive use, and early sexual intercourse before age 18.

It implied that the effectiveness of the health care programs and systems in society suffers from women’s characteristics. Furthermore, design planners and decision-makers should scale up health education opportunities in communities to change their attitudes about cultural beliefs and norms of early marriage and pregnancy.

## Abbreviations

CI: Confidence Interval
CSA: Central Statistical Agency
CDC: Centers for Disease Control and Prevention
EDHS: Ethiopia Demographic and Health Survey
EAs: Enumeration Areas
FMOH: Federal Ministry of Health
SDGs: Sustainable Development Goals
SNNPs: Southern Nation and Nationality Peoples
STIs: Sexual transmitted infectious
